# Stability of double-stranded oligonucleotide DNA with a bulged loop: a microarray study

**DOI:** 10.1186/2046-1682-4-20

**Published:** 2011-12-13

**Authors:** Christian Trapp, Marc Schenkelberger, Albrecht Ott

**Affiliations:** 1 Experimentalphysik, Universität des Saarlandes, D-66041 Saarbrücken, Germany

## Abstract

**Background:**

DNA is a carrier of biological information. The hybridization process, the formation of the DNA double-helix from single-strands with complementary sequences, is important for all living cells. DNA microarrays, among other biotechnologies such as PCR, rely on DNA hybridization. However, to date the thermodynamics of hybridization is only partly understood. Here we address, experimentally and theoretically, the hybridization of oligonucleotide strands of unequal lengths, which form a bulged loop upon hybridization. For our study we use in-house synthesized DNA microarrays.

**Results:**

We synthesize a microarray with additional thymine bases in the probe sequence motifs so that bulged loops occur upon target hybridization. We observe a monotonic decrease of the fluorescence signal of the hybridized strands with increasing length of the bulged loop. This corresponds to a decrease in duplex binding affinity within the considered loop lengths of one to thirteen bases. By varying the position of the bulged loop along the DNA duplex, we observe a symmetric signal variation with respect to the center of the strand. We reproduce the experimental results well using a molecular zipper model at thermal equilibrium. However, binding states between both strands, which emerge through duplex opening at the position of the bulged loop, need to be taken into account.

**Conclusions:**

We show that stable DNA duplexes with a bulged loop can form from short strands of unequal length and they contribute substantially to the fluorescence intensity from the hybridized strands on a microarray. In order to reproduce the result with the help of equilibrium thermodynamics, it is essential (and to a good approximation sufficient) to consider duplex opening not only at the ends but also at the position of the bulged loop. Although the thermodynamic parameters used in this study are taken from hybridization experiments in solution, these parameters fit our DNA microarray data well.

## Background

The hybridization process - the formation of the well-known double-helix structure from two complementary nucleic acid strands (such that A · T and C · G base pairs are formed) - is pivotal to the living organism. Among other important biotechnological methods, PCR or DNA microarray technology rely on it.

DNA microarrays consist of regular spaced domains of surface-attached probe sequences, which act as binding sites for their complementary fluorescently-labeled target sequences in solution. The probe sequence and position of each domain on the surface is known and the amount of bound target DNA can be determined quantitatively. Microarrays are important in many biotechnological methods such as gene expression profiling, where complex target oligonucleotides mixtures need to be analyzed in a highly parallel manner [1-3].

Due to the very sensitive molecular recognition process of DNA, one is in principle able to detect even small sequence deviations with the help of DNA microarrays. However, DNA targets that are not perfectly complementary can also form duplexes with the surface bound probes, albeit are less stable than the perfectly matching correspondent (PM).

Although DNA microarrays are widely used in biological and biotechnological applications, the underlying physical mechanisms of the hybridization process are poorly understood. Data analysis is mostly based on empirical, statistical methods [4-6]. To fully exploit the potential of the DNA microarray technology, it is desirable to pursue a more fundamental approach to the stability of hybridized or partly hybridized strands. Molecular simulations have greatly increased our understanding of DNA dynamics, thermal fluctuations and hybridization. DNA hybridization and mechanical properties of DNA e.g. the persistence length in the presence of surfaces were investigated on the molecular level [7-9]. While molecular simulations give a very detailed view of the molecular dynamics, here we are interested in a simple scheme to assess the stability of bulged loops on a DNA microarray. Systematic experiments on short bulged loops have hardly been performed.

The standard model for hybridization in solution is the so called two-state-nearest-neighbor model (NN-Model), which treats the formation of the DNA duplex as a two-state process where the duplex is either fully hybridized or fully denatured [10,11]. The model calculates the binding free energy of a perfectly complementary double-stranded duplex by summing the nearest-neighbor interaction parameters (10 experimentally determined free energy parameters [12-14]). These parameters take into account, that DNA stability arises from hydrogen bonding and base stacking interactions. Furthermore it is possible to extend the model and include single base mismatch (MM) defect parameters [13]. This model proved very successful for the prediction of duplex melting temperature *T_m _*in solution.

In several experiments, incorporated MMs have had a position dependent influence on the fluorescent signal. Zhang *et al*. suggested the position-dependent-nearest-neighbor-model (PDNN) [15] where the binding free energy of the duplex is calculated as a weighted sum of the nearest-neighbor parameters. The weight parameters are determined empirically.

In the past we experimentally and theoretically investigated the effect of single MMs on the duplex stability of a DNA microarray in the case where the lengths of probe and target match [16-18]. We have shown that a two state NN-model could not predict the MM binding affinities precisely. Therefore we developed a different theoretical approach, based on a double-ended molecular zipper [19-21]. The double-ended molecular zipper considers, that the duplex can only open from the ends. This simplification is justified because base pairs, which are located away from the duplex ends are less stable. This holds even if a single MM is incorporated into the duplex. Taking into account the heterogeneity of the binding affinities due to synthesis defects, the DNA microarray data could be reproduced with the model. We have shown that the double-ended zipper model maps to the PDNN model, while the former is derived from first principles [16]. The purpose of this study is to investigate the case where probe and target have unequal lengths and bulged loops form upon hybridization. Bulged loops are referred to as loops in the following. With our DNA microarray setup, loops of different lengths and at different positions can be obtained in a controlled manner by inserting additional bases into the perfectly matching probe sequence. The formation of loops increases the complexity of the hybridized state: new binding states between probe and target strands may emerge. We show that a good reproduction of the experimental data remains possible with the molecular zipper, but only if duplex opening can also occur at the loop position.

## Methods

### DNA Microarray Hybridization Experiments

We use in-house synthesized DNA Microarrays. All employed protocols including the preparation of dendrimer-functionalized microarray substrates, the light-directed synthesis (a "maskless" photolithographic technique based on NPPOC-phosphoramidites), as well as the data analysis methods are provided in Naiser *et al*. [18]. The only difference to the previously published experimental setup is a more homogeneous illumination of the microarray surface as well as an increased resolution due to the improved optics.

To avoid target-target interaction and competitive hybridization effects, only one target species (see table 1) is employed in the hybridization experiments. Probes on the microarray surface are coupled to the surface with their 3'-end. Hybridization temperature is 317 K.

**Table 1:** Target sequence Cy3-labeled perfect matching target sequence in solution.

Target sequence	length(bases)
5'-AAGTTATGATGAGTATTAATGGTTGTTGTAATG-3'	33

Images of the hybridized DNA microarray are taken for data analysis after thermal equilibrium is reached.

In order to test if the microarray surface has a significant influence on the hybridization, we repeat our experiments with the reversed probe sequence. The 5'-end of the sequence employed throughout this work corresponds to the 3'-end of the test sequence. No influence of the microarray surface on the hybridization could be detected (see additional file 1: Influence of the microarray surface on the hybridization signal).

### Probe Design

**Figure 1:**
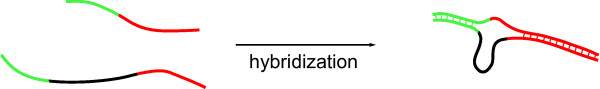
**Formation of a complementary strand by bulging of the non-complementary sequence**. The green and the red duplex parts of the two strands are of complementary sequence. Complete hybridization can occur only if the black portion forms a bulged loop.

To generate single-stranded DNA loops in the probe-target-duplexes, we introduce additional poly-T-sequences into the PM sequence. This is illustrated in Figure 1. The poly-T-sequence (black) is located between the red and the green parts of the probe strand. The green and red parts of the probe strand are complementary to the corresponding parts in the target strand. Upon hybridization, the black part forms a loop. By varying the length of the poly-T-sequence and the position at which the poly-T-sequence is introduced into the probe motif, generation of loops of different lengths and at different positions can be achieved.

**Figure 2:**
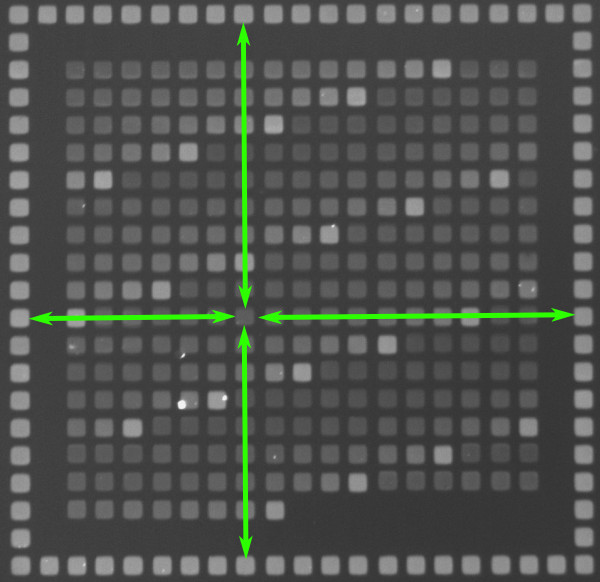
**Hybridization signals scaled with respect to PM grid**. The intensity of each feature inside the feature block can be normalized taking into account the average signal of four corresponding PM features (green arrows). With this data, illumination gradients are corrected linearly across the feature block.

In this way, poly-T-loops up to a length of 13 bases, at 20 different positions along the strand, amounting to 260 different probe sequences are generated during the *in situ *synthesis. To control the synthesis quality, 20 PM features are added. A single "feature block" consists of these 280 features organized as a square (see Figure 2). This feature block is synthesized 4 times on the microarray. Table 2 lists the synthesized probe sequences.

**Table 2:** Probe sequences

loop position	loop length	Probe sequence	length (bases)
PM Probe		3'-TTCAATACTACTCATAATTACCAACAACATTAC-5'	33

7	1	3'-TTCAATA**T**CTACTCATAATTACCAACAACATTAC-5'	34

7	2	3'-TTCAATA**TT**CTACTCATAATTACCAACAACATTAC-5'	35

.	.		.
.	.		.
.	.	3'TTCAATA**TT.**.CTACTCATAATTACCAACAACATTAC-5'	.

7	13	3'-TTCAATA**TTTTTTTTTTTTT**CTACTCATAATTACCAACAACATTAC-5'	46

8	1	3'-TTCAATAC**T**TACTCATAATTACCAACAACATTAC-5'	34

8	2	3'-TTCAATAC**TT**TACTCATAATTACCAACAACATTAC-5'	35

.	.		.
.	.		.
.	.		.

26	13	3'-TTCAATACTACTCATAATTACCAACA**TTTTTTTTTTTTT**ACATTAC-5'	46

Synthesized probe sequences (features) on the DNA microarray surface.

Bold letters highlight the additional thymine bases. Altogether, there are 261 different features.

We also synthesized probes with other loop sequences than the discussed poly-T. We investigated the influence of poly-C-sequences and random sequences on duplex stability as a function of loop length. The results are provided in additional file 2: Duplex stability of DNA duplexes with bulged loops of different sequences as a function of loop length. We didn't observe a significant change in the dependence of the fluorescent signal as a function of loop length as compared to the poly-T-sequences.

### Data Acquisition

**Figure 3:**
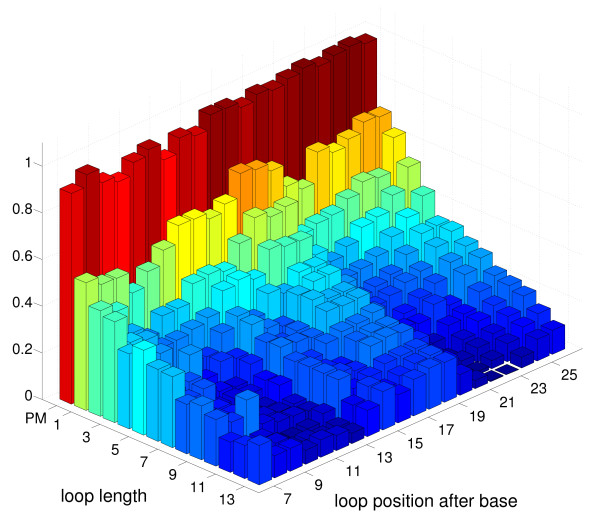
**Fluorescent signals as function of loop length and loop position**. Loop lengths *L *vary from 0 (PM) to thirteen additional thymine bases, loop positions *P *vary from 7 to 26. Loop position *P *means that the loop forming additional bases are inserted after base number *P *of the probe motif (counted from the surface). Strongest signal is set to 1, weakest signal is set to 0. Hybridization temperature is 317 K.

In order to determine the fluorescence intensities ("hybridization signals") of the microarray features from hybridized, fluorescently labeled target molecules, we take images of the DNA microarray surface with a fluorescent microscope. In Figure 2, we show such an image. A feature block (see Probe Design for definition) is surrounded by PM features. These PM features help control the illumination quality during synthesis and microscopic observation. For each feature inside the feature block, there are four corresponding PM features (green arrows). The average signal of these four PM features is used to correct the signal of the feature by normalizing the latter with respect to the average signal of the PM features. Synthesis-related illumination gradients can be - at least linearly - canceled out. To reduce experimental error, we reproduce the same feature block on the microarray at four different locations. To obtain the final data set, we take the average of the normalized signals of these four feature blocks. Error can be due to inhomogeneities of the microarray surface, fluorescent stains in the feature blocks or illumination gradients during the synthesis. The hybridization signals as a function of loop length and loop position of the final data set are shown in Figure 3. Hybridization temperature is 317 K. Strongest and weakest hybridization signals are normalized to 1 and to 0 respectively. For further details [18,22].

## Results and Discussion

### Binding Affinities as a Function of Loop Length

**Figure 4:**
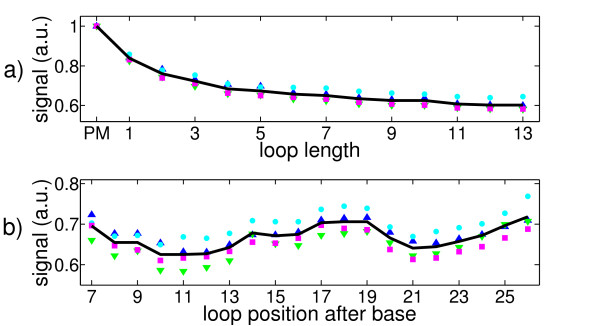
**Experimentally determined fluorescent signals**. Symbols: feature block 1, blue upward-pointing triangles; feature block 2, cyan circles; feature block 3, green downward-pointing triangles; feature block 4, magenta squares; average of all feature blocks, solid black line. **a) **Fluorescent signals as a function of loop length (from loop length 0 (PM), to loop length 13) averaged over all loop positions. PM signal is set to 1. **b) **Hybridization signals as a function of loop position averaged over all loop lengths. Loop position 7 indicates that the loop is inserted after base number 7 of the probe motif counted from the surface.

Figure 4a shows the dependence of the hybridization signal as a function of loop length averaged over all loop positions. The intensity of the PM is set to 1. We note a monotonic decrease of the signal with increasing loop length. The insertion of a single base already reduces the hybridization intensity to about 85% of the PM signal, 13 additional bases (largest number of additional bases under study) reduce the signal to about 60% of the PM signal. With a zipper, MMs in the middle of the duplex affect duplex stability most, because they are included in many of the possible states that are considered in the partition function. The employed probe strands are short compared to the length of DNA sequences used in other applications, which explains why the decrease in signal intensity after inserting a single additional base seems unusually strong.

#### Binding Affinities as a Function of Loop Position

Figure 4b shows the measured hybridization signals as a function of loop position in 3' to 5' direction after averaging over all loop lengths (PM signal set to 1). The resulting "loop position defect profile" is symmetric with respect to the center of the duplex. The signal is strongest for loops at the end, as well as in the middle of the duplex, it is weakest for loops at a distance of about 3-4 bases from the center. The difference between maximum and minimum is about 10% only. This is a weak variation compared to the hybridization signal as a function of loop length.

**Figure 5:**
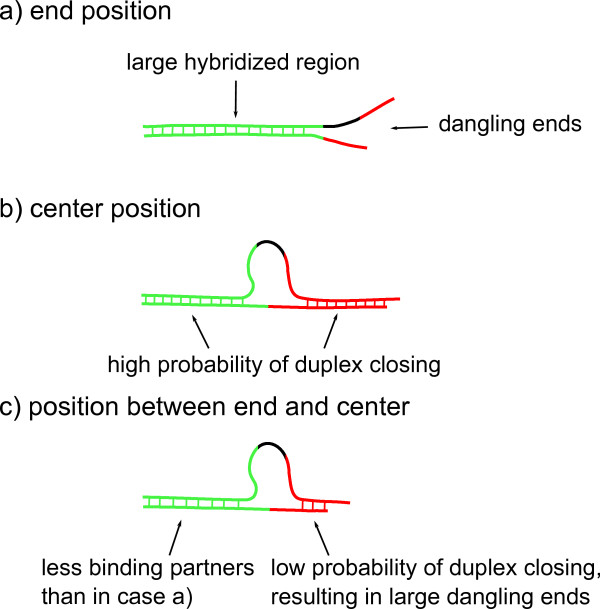
**Signal dependence on loop position**. **a) **A loop at a duplex end may result in dangling ends owing to the low probability of duplex closing. The large green region may provide high duplex stability and a strong fluorescent signal. **b) **If the loop is located towards the center of the duplex, it is likely that the duplex is closed at both sides of the loop due to the large number of possible binding partners on both sides of the loop. **c) **At a position located between end and center of the strand, the duplex is less stable than in a) and b).

From the following arguments, the dependence of the fluorescent signal on loop position, can be understood at least qualitatively (see Figure 5):

Loops positioned close to either end of a duplex have less potential binding sites towards that end, and they can open to form a dangling end. However, in this case a large part of the duplex to the opposite side of the loop remains strongly bound (Figure 5a).

Loops located at a center position have many possible binding partners to the left and to the right resulting in a closed loop and higher duplex stability (Figure 5b).

In between both of the extremes above, the hybridization signal drops to a minimum. This is because on one side these loops have less binding partners than loops in the middle of the duplex. On the other, the large hybridized part is shorter than for loops occupying end positions (Figure 5c).

### Thermodynamics of DNA Hybridization

At equilibrium single stranded probes *P *and target molecules *T *form a duplex *D *with a rate constant *k*_+_, they denature with a rate constant *k*_-_:

(1) $$P+T\overset{k_{+}}{\underset{k_{-}}{⇌}}D$$

This process can be described with a Langmuir-type adsorption isotherm. Since targets were in excess in our experiments, the target concentration [*T*] = [*T*_0_] is considered constant. The fraction of hybridized probes *θ*:

(2) $$\theta =\frac{\left[D\right]}{\left[P_{0}\right]}=\frac{K\cdot \;\left[T_{0}\right]}{1+K\cdot \;\left[T_{0}\right]}$$

where *K *is the equilibrium binding constant of the probe-target duplex. Since the fluorescent signal of the array is proportional to the fraction of hybridized probes *θ*, we think of *θ *as the "hybridization signal" in the following.

The Langmuir-type adsorption isotherm (2) has a very narrow transition region from low to high binding affinity. Our experimental data from previous experiments exhibits a broadened transition region. As we have shown [16,17], this is due to the heterogeneity of binding affinities due to unavoidable sequence defects during the *in situ *synthesis. It is necessary to describe the situation with a distribution of binding constants *K_i_*. Thus, the hybridization signal of an individual probe with random defects reads:

(3) $$\theta_{i}=\frac{K_{i}\cdot \;\left[T_{0}\right]}{1+K_{i}\cdot \;\left[T_{0}\right]}$$

Assuming that the synthesis defects follow a binominal distribution with a probability *p *that a defect occurs, the hybridization signal *θ *of a single feature is:

(4) $$\theta =\underset{k^{\prime }}{\sum }x_{k^{\prime }}\cdot \frac{\overset{\left(\begin{array}{c} N^{\prime }\\ k^{\prime } \end{array}\right)}{\underset{i=1}{\sum }}\theta_{i}}{\left(\begin{array}{c} N^{\prime }\\ k^{\prime } \end{array}\right)}=\underset{k^{\prime }}{\sum }x_{k^{\prime }}\cdot \frac{\overset{\left(\begin{array}{c} N^{\prime }\\ k^{\prime } \end{array}\right)}{\underset{i=1}{\sum }}\frac{K_{i}\cdot \;\left[T_{0}\right]}{1+K_{i}\cdot \;\left[T_{0}\right]}}{\left(\begin{array}{c} N^{\prime }\\ k^{\prime } \end{array}\right)}$$

*N *' is the number of bases in the probe, *k*' the number of synthesis defects and *x_k_*_' _is the probability that *k*' synthesis defects occur in a probe of length *N *'.

(5) $$x_{k^{\prime }}=\left(\begin{array}{c} N^{\prime }\\ k^{\prime } \end{array}\right)\cdot p^{k^{\prime }}\cdot {\left(1-p\right)}^{N^{\prime }-k^{\prime }}$$

To minimize computation time, synthesis defects are only considered up to a certain maximum number per strand $k_{max}^{\prime }$. The bases of the loop are treated synthesis defect free. Since the bases in the loop are, most of the time, only weakly or not at all bound (there are almost no complementary bases in the target strand), the consideration of synthesis defects in the loop is not necessary.

In our case, *N *' = 33, we took up to $k_{max}^{\prime }=3$ synthesis defects into account. This generates 6018 different probe sequences. Strands with more than 3 synthesis defects can be neglected (see additional file 3: Influence of the number of MMs on the fluorescent signal).

In the following, we calculate the binding constants *K_i _*as a function of loop position *P *and loop length *L *in thermodynamic equilibrium.

### Partition Function of the Double-ended Zipper

The partition function of the double-ended zipper model is [19-21]:

(6) $$Z_{D}=\overset{N-1}{\underset{k=0}{\sum }}\overset{N}{\underset{l=k+1}{\sum }}\omega_{k,l}=\overset{N-1}{\underset{k=0}{\sum }}\overset{N}{\underset{l=k+1}{\sum }}e^{\Delta G_{k,l}^{\circ }∕RT}$$

Here, *N *is the number of NN pairs and *ω_k,l _*is the statistical weight of the partially denatured state *S_k,l_*. *k *and *l *are the positions of the left and right zipper fork respectively. $\Delta G_{k,l}^{\circ }$ is the sum of NN free energies $\Delta g_{i}^{\circ }\;\left(\Delta g_{i}^{\circ }>0\right)$ of the zipped duplex sections.

(7) $$\Delta G_{k,l}^{\circ }=\left(\overset{l}{\underset{i=k}{\sum }}\Delta g_{i}^{\circ }\right)+\Delta g_{init}$$

Δ*g_init _*= -4.5 kcal/mol is the duplex initialization free energy [17]. For the binding constant *K_i _*(*P*, *L*), *K_i _*(*P*, *L*) = *Z_D _*(*P*, *L*) taking the totally denatured state, *S*_0_, as the reference state.

**Figure 6:**
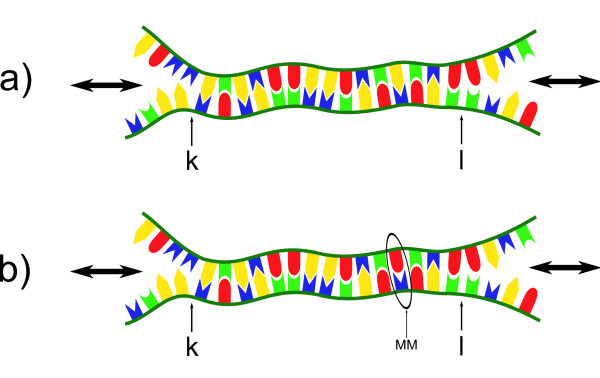
**Double-ended zipper model of DNA hybridization**. **a) **Duplex can only open and close from the ends in a zipper-like fashion. Between the two zipper fork positions *k *and *l*, the duplex is closed. $\Delta G_{k,l}^{o}$ is the sum over all closed NN parameters in the bound duplex section. **b) **One MM affects two NN pairs.

Figure 6 illustrates the double-ended zipper model and the corresponding notation. The duplex is hybridized between the zipper forks at positions *k *and *l*. This corresponds to the free energy $\Delta G_{k,l}^{\circ }$. Duplex opening and closing occurs only at the ends indicated by the black arrows left and right to the duplex. In Figure 6b, a single MM is incorporated into the duplex.

#### Loop energy penalties

We have shown that it is sufficient to include MM defect parameters into a zipper model to account for single base defects [17]. In the following, we test this simple model for the case of loops. For single stranded DNA loops we calculate purely entropic energy penalties by treating the DNA loop as a self-avoiding random walk (SAW) on a lattice. Since duplex opening can only occur from the ends and therefore the DNA loops are always closed, only SAWs which return to the origin need to be considered. For the number of SAWs of length *l *returning to the origin in the limit *l *→ ∞ [23,24]:

(8) $${\#}_{origin}\left(l\right)\propto \sigma \cdot \frac{\mu^{l}}{l^{c}}$$

*σ *= 1, 75 · 10^-4 ^is the so-called cooperativity parameter, *μ *is the connectivity constant and *c *= 2, 15 is the loop closure exponent. *σ *and *c *are universal constants whereas *μ *(*μ *= 4, 684 used here) depends on the considered geometry.

For the total number of SAWs of length *l *of all possible SAW configurations [23,25]:

(9) $${\#}_{total}\propto \mu^{l}\cdot l^{\gamma -1}$$

*γ *= 1, 157 ± 3.10^-3 ^is the (universal) entropic exponent. That gives us the probability *ρ *that a SAW of length *l *returns to the origin:

(10) $$\rho \left(l\right)=\frac{{\#}_{origin}}{{\#}_{total}}\propto \frac{\sigma \cdot \frac{\mu^{l}}{l^{c}}}{\mu^{l}\cdot l^{\gamma -1}}$$

Given *ρ*(*l*), we can calculate the entropy *S*(*l*) and the corresponding loop energy penalties Δ*G_entropy_*(*l*):

(11) $$\begin{array}{ll} \;S\left(l\right)\;\;\;\; & =N_{A}\cdot k_{B}\cdot ln\left[\rho \left(l\right)\right]\;\\ \Rightarrow \Delta G_{entropy}\left(l\right) & =-T\cdot S\left(l\right)\;\\ & \; \end{array}$$

The length of a DNA loop is determined by the number of bases *L *in the loop and the distance *a*_0 _between two adjacent bases. The length of a random walk is the number of steps from start to end on a lattice with the lattice parameter *p*_0_. When treating a DNA loop as a SAW, one has to consider the persistence length of single stranded DNA, which determines the number of steps in the SAW and defines the lattice parameter *p*_0_. Since *p*_0 _and *a*_0 _rank in the same dimension depending on the salt concentration [26-28], we take a DNA loop of length *L *· *a*_0 _as a SAW with *L *steps on a lattice with the lattice parameter *p*_0 _≈ *a*_0 _(salt concentration is 0.90 M NaCl and 50 mM NaH_2_PO_4_). Moreover, we test the influence of *p*_0 _on the absolute loop energy penalties values Δ*G_entropy_*. This shows that the differences are negligible. This means:

(12) $$\Delta G_{entropy}\left(L\right)=-T\cdot S\left(L\right)$$

where *L *ranges from 1 to 13.

**Figure 7:**
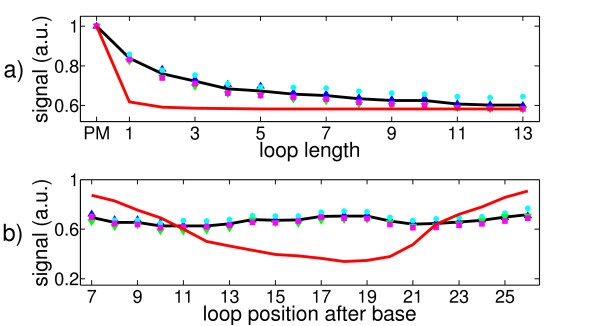
**Experimental data and theoretical signals calculated with a double-ended zipper (no opening at loop position)**. Symbols: feature block 1, blue upward-pointing triangles; feature block 2, cyan circles; feature block 3, green downward-pointing triangles; feature block 4, magenta squares; average of all feature blocks, solid black line; calculated signals, solid red line. **a) **dependence of experimentally observed fluorescent signals on loop length after averaging over all loop positions compared to the calculated fluorescent signals averaged over all loop positions. **b) **dependence of experimentally observed fluorescent signals on loop position after averaging over all loop lengths compared to the calculated fluorescent signals averaged over all loop lengths.

Figure 7 shows the comparison between our experimental signals and predictions with the simple zipper model. a) Hybridization signals as a function of loop lengths averaged over all loop positions. b) Hybridization signals as a function of loop position averaged over all loop lengths. The symbols indicate the signals of each feature block, the solid black line is the average signal of all feature blocks and the red solid line represents the theoretically predicted signals. The experimental results cannot be reproduced based on the simple zipper.

### Extension of Double-ended Zipper Model

So far duplex opening was only possible from the ends of the duplex. States, in which the duplex zips at the loop position, are essential for the correct reproduction of our experimental results.

The partition function of a duplex *Z_on _*(*P*, *L*) as a function of loop position *P *and loop length *L *can be decomposed as a sum of five elements:

*Z_zipper _*(*P*, *L*): The hybridized strands zip from both ends. Partition function as presented in the section above.

**Figure 8:**
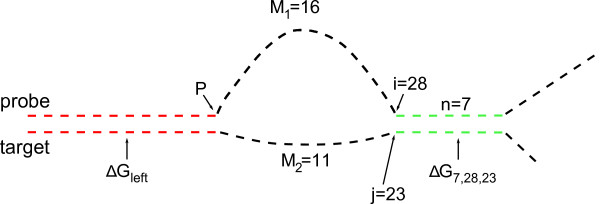
**Notation of. Z_extended,right_**. Here, each line represents one base, a loop of length *L *= 9 (nine additional thymine bases) is inserted after base number 12 of the PM probe motif (loop position *P *= 12). The red lines represent hybridized duplex parts (Watson-Crick base pairing) and black lines denatured parts (the black lines in the middle of the probe include the additional loop bases). To the right of the loop position *P*, probe and target strand can bind in every possible conformation to each other resulting in Watson-Crick and non-Watson-Crick base pairing (green lines). The free energy of this part of the duplex is accounted for by Δ*G*_7,28,23_. In this way, bulged loops form in probe and target strand of different lengths. Here, a loop of length *M*_1 _= 16 bases and *M*_2 _= 11 bases is formed in probe and target respectively. Both loops reunite after base number *i *= 28 of the probe and base number *j *= 23 of the target strand. The black, rightmost part of the duplex is denatured and not considered in the computation. To reduce computing time, the duplex part left of the loop position is considered hybridized all the time. The free energy Δ*G_left _*of this part is calculated with the help of the zipper model (6).

*Z_extended,right _*(*P*, *L*): Probe and target strands right of the loop position can undergo every possible binding configuration among each other (not limited to a zipper). Thus, loops of different size in probe and target strand can appear. The duplex part left of the loop position zips to and from the loop position. The free energy of this part is considered in Δ*G_left_*. Figure 8 illustrates *Z_extended,right_*. The red and green dashed lines represent hybridized duplex parts. The black dashed lines are denatured (at the end) or they form a loop between probe and target (middle). Here in this particular case, the probe and target strand form a loop of 16 and 11 bases respectively. The two strands reunite after base 28 of the probe strand and base 23 of the target strand, the following 7 bases are hybridized. This results in the free energy Δ*G*_7,28,23_.

*Z_extended,left _*(*P*, *L*): analogous to

*Z_extended,right _*(*P*, *L*) but opposite side.

*Z_double zipper _*(*P*, *L*): Both parts, left and right from the loop position behave like an independent zipper. To avoid double count of states from adding *Z_extended,right _*(*P*, *L*) and *Z_extended,left _*(*P*, *L*), this partition function needs to be subtracted.

*Z_non-canonical _*(*P*, *L*): This partition function sums over all non-canonical binding states which occur simultaneously on both sides of the loop position. As we show below, this term can in principle be neglected because all of these binding states bind only weakly.

In the full expression for *Z_extended,right _*the summation of all possible binding configurations between both strands right of the loop position depends on the zipping state *S_k,l _*of the duplex part left of the loop position. This makes the calculation computation intensive. To reduce computing time of the model, we use *Z_extended,right _*approximated by (Figure 8):

(13) $$\begin{array}{c} Z_{extended,right}\left(P,L\right)=\overset{N-P+1}{\underset{n=2}{\sum }}\overset{N+L-n+1}{\underset{i=P}{\sum }}\overset{N-n+1}{\underset{j=P}{\sum }}\omega_{n,i,j}\\ \text{with}\;\;\omega_{n,i,j}=e\frac{\Delta G_{n,i,j}+\Delta G_{entropy,right}+\Delta G_{left}}{RT}\\ \text{and}\;\;\Delta G_{n,i,j}=\overset{n-1}{\underset{r=1}{\sum }}\Delta g_{r}^{ij} \end{array}$$

*i *and *j *mark the positions of the zipper forks in probe and target respectively, *n *bases of probe and target strand (*n *- 1 NN pairs) of the region to the right of *i *and *j *are hybridized. Thus, Δ*G_n,i,j _*is the NN energy of (*n *- 1) base pairs which are hybridized from zipper fork position *i *in the probe and zipper fork position *j *in the target. $\Delta g_{r}^{ij}$ is the NN energy of a single hybridized base pair in this region.

The free energy of the duplex part left of the loop position Δ*G_left _*is approximated using the zipper model (6). Since Δ*G_left _*is a function of the loop position *P *only, and it is independent from the current zipping state *S_k,l_*, computing time is greatly reduced.

(14) $$\begin{array}{c} \Delta G_{left}=\Delta G_{left}\left(P\right)=RT\cdot ln\left[\overset{P-2}{\underset{k=0}{\sum }}\overset{P-1}{\underset{l=k+1}{\sum }}\omega_{k,l}\right]\\ \text{with}\;\omega_{k,l}\;\text{defined}\;\text{as}\;\text{before}\text{.} \end{array}$$

We calculate the binding constants

(15) $$\begin{array}{ll} K_{i}\left(P,L\right) & =Z_{zipper}+Z_{extended,right}\;\\ & +Z_{extended,left}-Z_{double\;zipper}\;\\ & \; \end{array}$$

with and without our approximation (13) for *Z_extended,right_*(*P*, *L*) (and (20) for *Z_extended,left_*(*P*, *L*) below) for one duplex sequence. To fit the theoretical signals to the experimental data we use a scaling factor *C*, which links the calculated binding constants to the fluorescent intensity values (*C *is a free parameter):

(16) $$\theta_{i}=\frac{C\cdot K_{i}\cdot \left[T_{0}\right]}{1+C\cdot K_{i}\cdot \left[T_{0}\right]}$$

**Figure 9:**
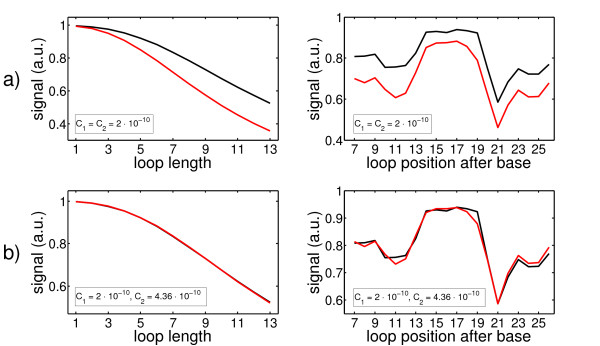
**Comparison between the signals calculated with and without approximation for Z_extended,right _and Z_extended,left_**. Symbols: signal calculation without approximation, black solid line; signal calculation wit approximation red solid line. We compare the calculated fluorescent signals resulting from *K_i _*for a duplex sequence without synthesis defects to test our approximation for *Z_extended,right _*and *Z_extended,left_*. **a) **If the same scaling factor *C *= *C*_1 _= *C*_2 _is used, the two curves differ in absolute values, but the shape is very similar. **b) **By choosing two different scaling factors *C*_1_,*C*_2_, the two curves overlap very well. This shows that our approximation for *Z_extended,right _*and *Z_extended,left _*is excellent.

Figure 9 shows that our approximation for *Z_extended,right _*(13) and for *Z_extended,right_*(*P*, *L*) (20) is excellent, if *C *is adjusted properly. If the factor *C *is the same for the calculation with and without approximation, the red and black curve differ in absolute values but the shape remains very similar (left side in Figure 9). By adjusting *C*, the two curves overlap (right side). The reason for this is that the approximations (13) and (20) neglect some binding states resulting in (slightly) smaller overall binding constants.

In the extended model, it is possible that loops start at some origin $\vec{0}$ and end at position $\vec{r}$. Now we obtain for two SAWs with *M*_1 _and *M*_1 _steps:

(17) $$\begin{array}{lll} \Delta G_{entropy,right} & =\Delta G_{entropy,right}\left(M_{1},M_{2}\right)\;\\ & =-k_{B}T\cdot ln\left[\rho \left(M_{1},M_{2}\right)\right]\;\\ \text{with}\;M_{1} & =i-P\;\\ \text{and}\;M_{2} & =j-P\;\\ & \; & \text{(5)} \end{array}$$

*ρ*(*M*_1_, *M*_2_) is the probability that two SAWs with number of steps *M*_1 _and *M*_2 _respectively start at the origin and meet again. Here, we have:

(18) $$\begin{array}{c} \rho \left(M_{1},M_{2}\right)=\underset{\vec{r},{\vec{r}}^{\prime }}{\sum }\delta \left(\vec{r}-{\vec{r}}^{\prime }\right)\cdot \frac{\#\left(M_{1},\vec{r}\right)\cdot \#\left(M_{2},{\vec{r}}^{\prime }\right)}{{\#}_{total}\left(M_{1}\right)\cdot {\#}_{total}\left(M_{2}\right)}\\ =\underset{\vec{r}}{\sum }\frac{\#\left(M_{1},\vec{r}\right)\cdot \#\left(M_{2},\vec{r}\right)}{{\#}_{total}\left(M_{1}\right)\cdot {\#}_{total}\left(M_{2}\right)}\\ \text{with}\;\left|\vec{r}\right|\leq min\left(M_{1},M_{2}\right) \end{array}$$

$\#\left(M_{i},\vec{r}\right)$ is the number of SAWs with *M_i _*steps which start at the origin and end at position $\vec{r}$. In 3D [23]:

(19) $$\begin{array}{c} \#\left(M_{i},\vec{r}\right)\propto \mu^{M_{i}}\cdot M_{i}^{\gamma -1-3\nu }\cdot g\left(\frac{r}{M_{i}^{\nu }}\right)\\ \text{with}\;g\left(x\right)\propto x^{\varphi }\cdot e^{-\lambda x^{\delta }}\text{, }\lambda >0\text{,}\delta =\frac{1}{1-\nu }\\ \text{and}\;\varphi =\frac{\gamma -1}{\nu } \end{array}$$

Constants *γ *and *μ *are defined as before. *ν *= 0, 588 ± 1, 5 · 10^-3 ^is the (universal) metric exponent. #*_total_*(*M_i_*) is the total number of SAWs of *M_i _*steps (as defined in equation (9)).

In an analogous manner, *Z_extended,left _*is calculated:

(20) $$\begin{array}{c} Z_{extended,left}\left(P,L\right)=\overset{P}{\underset{n=2}{\sum }}\overset{L+P-n}{\underset{i=0}{\sum }}\overset{P-n}{\underset{j=0}{\sum }}\omega_{n,i,j}\\ \text{with}\;\omega_{n,i,j}=e^{\frac{\Delta G_{n,i,j}+\Delta G_{entropy,left}+\Delta G_{right}}{RT}}\\ \text{and}\;\Delta G_{n,i,j}=\overset{n-1}{\underset{r=1}{\sum }}\Delta g_{r}^{ij} \end{array}$$

Here we have

(21) $$\begin{array}{cc} \Delta G_{right} & =\Delta G_{right}\left(P\right)\\ & =RT\cdot ln\left[\overset{N-1}{\underset{k=P}{\sum }}\overset{N}{\underset{l=k+1}{\sum }}\omega_{k,l}\right] \end{array}$$

and finally

(22) $$\begin{array}{ll} \Delta G_{entropy,left}\left(M_{1},M_{2}\right) & =-k_{B}T\cdot ln\left[\rho \left(M_{1},M_{2}\right)\right]\;\\ \text{with}\;M_{1} & =L+P-n-i\;\\ \text{and}\;M_{2} & =P-n-j\;\\ & \; \end{array}$$

For *Z_double zipper_*(*P*, *L*), we have:

(23) $$\begin{array}{ll} Z_{double\;zipper}\left(P,L\right) & =\underset{k,l}{\sum }\underset{o,p}{\sum }\omega_{k,l,o,p}\;\\ \text{with}\;\omega_{k,l,o,p} & =e^{\frac{\Delta G_{k,l}+\Delta G_{entropy,double}+\Delta G_{o,p}}{RT}}\;\\ & \; \end{array}$$

And Δ*G_entropy,double_*:

(24) $$\begin{array}{ll} \Delta G_{entropy,double}\left(M_{1},M_{2}\right) & =-k_{B}T\cdot ln\left[\rho \left(M_{1},M_{2}\right)\right]\;\\ \text{with}\;M_{1} & =o-l+L-1\;\\ \text{and}\;M_{2} & =o-l-1\;\\ & \; \end{array}$$

In the case where probe and target length match, duplex zipping can only occur if the two strands are perfectly aligned. We consider the initiation energy, the entropic barrier to meet this constraint, as constant. We simply write *K_i _*= *Z_D _*and include the initiation energy in a prefactor.

In the case of duplexes with loops, the probe-target length difference Δ*L *increases the possible conformations of the probe strand, that do not promote duplex initiation. The initiation energy changes accordingly. Neglecting unfolding of the coils for duplex formation, the number of pairing collisions, that do no lead to zipping, grows linearly with Δ*L*, resulting in an initiation entropy change

(25) $$\Delta S_{init}\propto ln\left(1+\frac{\Delta L}{L_{0}}\right)$$

*L*_0 _is the characteristic length of the problem, which is the persistence length (in our experimental conditions this corresponds to a single base). In the case of a short, loop-forming sequence located in the center of the strand, however, there are two positions, where parallel but shifted probe and target strands can initiate duplex formation. These positions correspond to the matching sequence left and the right from the loop implying a correction of Δ*L*/*L*_0 _by 1/2. However, if the loop forms towards the ends, we are close to the situation of a single strand above. In the following we neglect this dependence on loop position and use a factor 1/2 throughout. Either factor (1 or 1/2) does not drastically modify our result, if the factor *C *is adjusted accordingly.

Our approximation for Δ*S_init _*tends to overestimate the corresponding initiation energy penalty as Δ*L *increases. This is because for large Δ*L *the situation differs: in this case the separated matching sequences are almost independent and the initiation energy tends to its asymptotic value of two independent hybridization events. As a conclusion for large Δ*L *a weaker dependence of the initiation energy on Δ*L *can be expected.

From (25), we get the modified binding constant *K_i_*:

(26) $$K_{i}\left(P,L\right)=\frac{Z_{D}\left(P,L\right)}{1+\frac{\Delta L}{2L_{0}}}$$

The calculation of the hybridization signal is then straight forward.

We note, that the choice of the denominator of equation (26) following from (25) has an impact on the calculated hybridization signals. Our theory could possibly be improved by choosing a different denominator which, however, may be a subtle problem by itself, not the scope of this paper.

**Figure 10:**
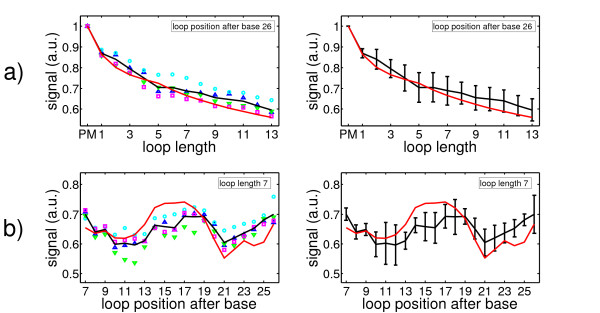
**Comparison between the experimentally observed fluorescent signals and the calculated affinities**. Symbols: feature block 1, blue upward-pointing triangles; feature block 2, cyan circles; feature block 3, green downward-pointing triangles; feature block 4, magenta squares; average of the feature block signals, solid black line; prediction, solid red line. **a) **The predicted and experimentally obtained hybridization signals as a function of loop length for one specific loop position. PM signal is set to 1. **b) **The dependence of the theoretical and experimental hybridization signals on loop position for one specific loop length. PM signal is set to 1. The figures to the right in a) and b) show the experimental average (black) with 95% confidence intervals and the predicted signals (red). The experimentally observed hybridization signals are reproduced well by our theory.

Figure 10 shows the comparison between our experimental results and theory. a) Hybridization signals as a function of loop length for one specific loop position. b) Hybridization signals as a function of loop position for one specific loop length. In the figures to the right, we give the 95% confidence intervals for our data points (black) and compare them to our theory (red). This shows that the experimentally observed trends and the reproduction with our model are statistically relevant. The different symbols indicate the signals of the different feature blocks as a function of loop position or length, the solid black line is the experimental average and the red solid line represents the theoretical predictions.

**Figure 11:**
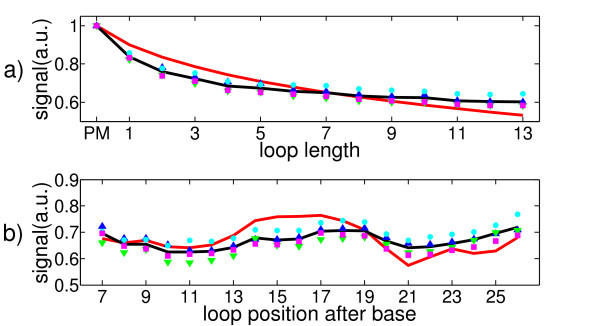
**Comparison between experiment and theory after averaging over loop length and loop position respectively**. Symbols: feature block 1, blue upward-pointing triangles; feature block 2, cyan circles; feature block 3, green downward-pointing triangles; feature block 4, magenta squares; average of all feature blocks, solid black line; prediction, solid red line. **a) **After averaging over all loop positions, the calculated and experimental hybridization signals as a function of loop length. PM signal is set to 1. **b) **The dependence of the calculated and experimental hybridization signals on loop position after averaging over all loop lengths. PM signal is set to 1. The experimentally observed hybridization signals are reproduced well by our theory.

To make the signal dependence on loop length clearer, we present the hybridization signals averaged over all loop positions as a function of loop length and compare them to the predicted signals (upper part of Figure 11). The lower part of the same figure shows the signal dependence as a function of loop position after averaging over all loop lengths. The symbols represent the signals of the feature blocks, the solid black line the average signal of all feature blocks and the solid red line represents the predicted signals.

Figure 10 and 11 show that the model reproduces our experimental findings well. Parameters used here were: simulation temperature *T_sym _*= 317 K, synthesis error rate *p = *0.084, energy penalty for synthesis related defects Δ*g_def,syn _*= -1 kcal/mol (consideration up to three errors per probe during synthesis, Δ*g_def,syn _*was determined in [16]). We use the temperature adjusted NN and MM defect parameters from [12,13] and the references therein. Since MM defect parameters are only available for isolated MMs, we include another parameter *MM_def _*= -2 kcal/mol for the case of two adjacent MMs (we approximate two adjacent MMs as two independent synthesis defects next to each other, therefore: *MM_defect _*= 2.*g_def,syn_*. Furthermore, we use the (universal) parameters for a SAW [23]. The only free parameters are the factor *C *= 1.5 · 10^-3 ^that links the theoretical binding constants *K_i _*to fit our experimental data of fluorescent signals and the probability for synthesis related defects *p *= 0.084 (the latter is not completely free since it is used to check if our theory is consistent with the coupling and deprotection efficiency of the used oligonucleotides).

We note that the partition function *Z_double zipper_*(*P*, *L*) alone already reproduces the approximate shape of the symmetric loop defect profile as shown in Figure 11b (dependence of the hybridization signal on loop position). However, the resulting binding constants are smaller than the ones calculated with *Z_extended,left _*and *Z_extended,right _*respectively. *Z_extended,left _*and *Z_extended,right _*help in reproducing the shape and moreover, the absolute values of the experimental signals (see additional file 4: Hybridization signals resulting from *Z_double zipper _*and comparison to *Z_extended,right _+ Z_extended,left_*).

Small differences between theoretical and experimental results regarding the signal dependence on loop position can be explained by the particularities of the duplex sequence under study. Here we look at two differences:

region ranging from loop position 14 to 18: this duplex region has many A/T bases and the distance between two C bases is the largest for the whole sequence. The duplex destabilization of an A/T rich region may be underestimated.

loop position 21: the region has many C bases and the loop bases are inserted after two existing C bases. It has been shown [22,29,30], that degenerated base pairs may reinforce binding considerably. Stabilization by degenerated base pairs is not included in our theory.

Although there are differences between experiment and theory, the deviations are small (see Figure 10 and 11). An even better agreement could be obtained by choosing a different dependence of the duplex initiation energy on Δ*L*. Our approximation for it (see above) only holds for short Δ*L*and we suppose the systematic deviation visible in Figure 11a from theory and experiment to originate from our approximation. As expected, at longer Δ*L*, we tend to underestimate the binding constant. To our knowledge, although an often encountered problem, no simple scheme to assess the initiation energy is known. Working out the dependence of the initiation energy between the two regimes discussed above (short and very long Δ*L*) is beyond the scope of this paper. Molecular simulations could help to provide better understanding of the nucleation process [7].

In literature, internal DNA loops or bubbles of total length *l *= *l*_1 _+ *l*_2 _e.g. occurring in DNA denaturation experiments are often treated as SAWs of the same length returning to their origin (*l*_1 _: unbound bases in probe; *l*_2 _: unbound bases in target) [24]. Reproduction of our experimental data could not be achieved when the calculation is done in this way, because the calculated loop energy penalties were much too large. Treating a DNA loop as a SAW of length *l *= *l*_1 _+ *l*_2 _returning to the origin is different from calculating the probability that two SAWs of given lengths *l*_1 _and *l*_2 _start at the same point and meet again at some distance. In the first case, the number of possible conformations is much higher because the constraint is weakened to any pair $l_{1}^{\prime },l_{2}^{\prime }$ with $l_{1}^{\prime }+l_{2}^{\prime }=l$, not just the given *l*_1_, *l*_2_. The first case could give the same same results if the calculation is done under the constraint that the loop of length *l *= *l*_1 _+ *l*_2 _reaches the position $\vec{r}$ where the two loops reunite after *l*_1 _steps similar to the way described in [31].

This may not always matter so much: the length of the probe sequences used throughout this study is much shorter than the length of DNA strands used in DNA denaturation experiments. Since the free energy of a short DNA strand is small, the size of the loop energy penalties is more crucial.

## Conclusions

In this paper we investigated the stability of DNA with a bulged loop. We inserted additional thymine bases into the surface-bound PM motif at a given position. By hybridizing DNA oligonucleotide targets onto the DNA microarray, bulged loops of different length and at different positions along the DNA duplex are formed.

We find that duplex stability decreases monotonically with the length of the bulged loop. Moreover, if the position of the bulged loop on the probe strand is varied, duplex stability exhibits a symmetric variation with respect to the center of the duplex. Duplex stability is highest for end- and middle-positions of the inserted bulged loop. For theoretical prediction we have shown that it is necessary and sufficient to consider strand opening at the position of the bulged loop. We have elaborated a successful approximation for the partition function of these new binding states. The signal dependence on loop length and on loop position could be reproduced with a limited amount of computing time (see Figure 11).

The employed NN free energy parameters from [12] are based on solution hybridization experiments. However, as we show in this study and in a previous paper [17], these parameters can be used to describe microarray hybridization well. The corresponding loop energy penalties can be obtained by considering the bulged loops as a self-avoiding walk on a lattice.

In our simulation, we use just two free parameters:

*C *= 1.5 · 10^-3^: scaling factor, which fits the calculated binding constants to the fluorescent light intensities. This parameter cannot be avoided.

*p *= 0.084: probability of a synthesis related defect. In a previous work, the value of *p *was determined to *p *= 0, 1. In our improved experimental setup, we have less stray light and a better resolution which result in a better synthesis quality (see Methods). Therefore, we chose *p *to be a free parameter. *p *is obtained as 0.084 in good agreement with the coupling and deprotection efficiency of the employed oligonucleotides and the achievable contrast of the optical setup [32-34]. Given this knowledge, p is not completely free and the resulting value is used to check the consistency of our theory.

The formation of bulged loops is an important aspect that needs to be considered when analyzing DNA microarray data or DNA hybridization of complex mixtures in general. Partly non-complementary sequences can form stable complementary duplexes through formation of a bulged loop resulting in false positive signals. The investigation of these bulged loop structures is therefore necessary to gain a deeper understanding of DNA hybridization and to make DNA microarrays and other, nucleic acid based high throughput technology based on DNA hybridization more reliable and accurate.

## Authors' contributions

CT carried out the experiments and statistical data analysis, computational modeling and drafted the manuscript. MS helped to draft the manuscript. AO conceived of the study, and participated in its design and coordination and aided in drafting the manuscript. All authors read and approved the final manuscript.

## Supplementary Material

Additional file 1 **Influence of the microarray surface on the hybridization signal**. We test the influence of the microarray surface on the hybridization signal by synthesizing probes with reversed sequence (3'-CATTACAACAACCATTAATACTCATCATAACTT-5'). The 5'-end of the sequence employed throughout this work corresponds to the 3'-end of the reversed sequence. No significant influence of the surface can be detected. Click here for file

Additional file 2 **Duplex stability of DNA duplexes with bulged loops of different sequences as a function of loop length**. Instead of the discussed poly-T loop sequences, we synthesize probes containing poly-C loop sequences and random loop sequences respectively at three different positions (the number of additional bases vary from one to thirteen; the random loop sequences are listed in table b) of this file). Upon hybridization with the target sequence listed in table 1, we note a monotonic decrease of the fluorescent signal as a function of loop length. After averaging over all loop positions, we compare the experimental signals as a function of loop length with the model predictions. We show that the experimental data is reproduced by our theory. Click here for file

Additional file 3 **Influence of the number of MMs on the fluorescent signal**. In order to reproduce our experimental data, it is sufficient to consider up to 3 synthesis-related defects in the zipper model. We confirm this by measuring the fluorescent signals of probes with 1 to 4 MMs. MMs are incorporated into the PM probe motif at 8 given positions resulting in 162 different probe sequences. To generate the MMs, we replace the bases at these specific positions with a thymine base (or with an adenine base, if a thymine base is already present at the specific position). After categorizing the probes into groups according to their number of MMs, we calculate the average signal of each group and plot it against the number of MMs (PM signal is set to 1, background signal is set to 0). Based on this data, we can estimate the error caused by neglecting probes with more than 3 synthesis defects: the error ≪ 4%, smaller than the experimental error. Click here for file

Additional file 4 **Hybridization signals resulting from Z_double zipper _and comparison to Z_extended,right _+ Z_extended,left_**. We compare the calculated hybridization signals resulting from *Z_double zipper _*to the signals resulting from. *Z_extended,right _*+ *Z_extended,left_*.The predicted hybridization signals are similar in shape but differ regarding absolute values. In the figure, the scaling factor *C*, which relates the predictions to the absolute signal intensities of the experiments, has been changed to 3 in case of *Z_double zipper_*, compared to *C *= 1.5 · 10^-3 ^throughout this study. Click here for file
