# Supplementary material for: Stability of double-stranded oligonucleotide DNA with a bulged loop: a microarray study
Source: BMC Biophys. 2011 Dec 13;4:20. doi: 10.1186/2046-1682-4-20 (PMC3262748; doi:10.1186/2046-1682-4-20)
Supplement: Additional file 1 — Influence of the microarray surface on the hybridization signal. We test the influence of the microarray surface on the hybridization signal by synthesizing probes with reversed sequence (3'-CATTACAACAACCATTAATACTCATCATAACTT-5'). The 5'-end of the sequence employed throughout this work corresponds to the 3'-end of the reversed sequence. No significant influence of the surface can be detected. [file 2046-1682-4-20-S1.PDF]

Supplementary Material
Additional File 1

"Stability of double-stranded oligonucleotide DNA with a bulged loop: a microarray study"

Christian Trapp, Marc Schenkelberger and Albrecht Ott

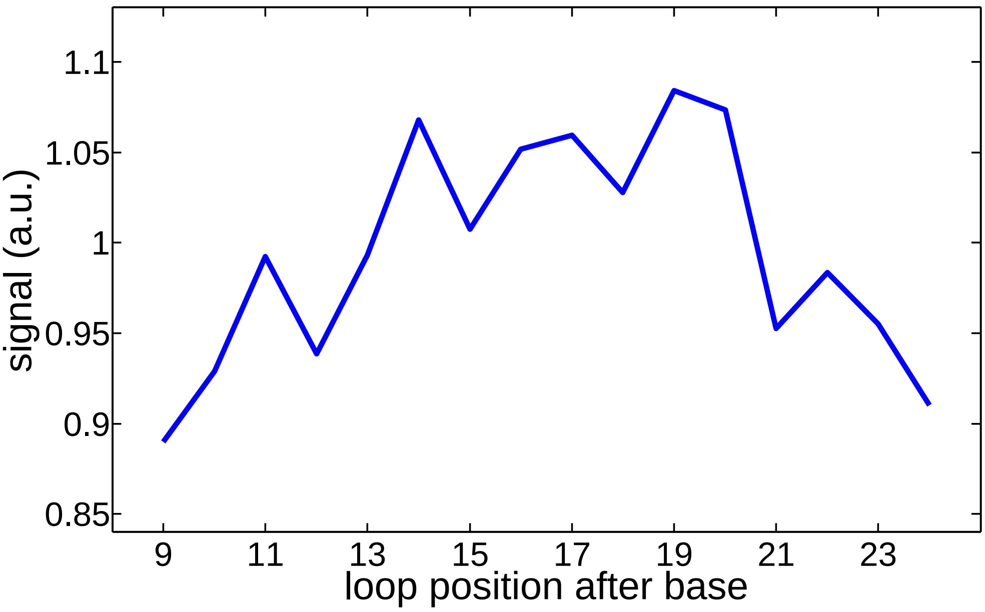

To test the influence of the microarray surface on the hybridization signal, we synthesized a DNA microarray with the reversed probe sequence 3'-CATTACAACAACCATTAATACTCATCATAACTT-5'. This produces no significant change. The figure shows the hybridization signals as a function of loop position averaged over all loop lengths for the reversed sequence. Hybridization temperature is 293 K. Loop position 9 indicates that the loop is inserted after base number 9 of the probe motif counted from the surface.
