# Supplementary material for: Stability of double-stranded oligonucleotide DNA with a bulged loop: a microarray study
Source: BMC Biophys. 2011 Dec 13;4:20. doi: 10.1186/2046-1682-4-20 (PMC3262748; doi:10.1186/2046-1682-4-20)
Supplement: Additional file 2 — Duplex stability of DNA duplexes with bulged loops of different sequences as a function of loop length. Instead of the discussed poly-T loop sequences, we synthesize probes containing poly-C loop sequences and random loop sequences respectively at three different positions (the number of additional bases vary from one to thirteen; the random loop sequences are listed in table b) of this file). Upon hybridization with the target sequence listed in table 1, we note a monotonic decrease of the fluorescent signal as a function of loop length. After averaging over all loop positions, we compare the experimental signals as a function of loop length with the model predictions. We show that the experimental data is reproduced by our theory. [file 2046-1682-4-20-S2.PDF]

Supplementary Material
Additional File 2

“Stability of double-stranded oligonucleotide DNA with a bulged loop: a microarray study”

Christian Trapp, Marc Schenkelberger and Albrecht Ott

Sequences of the bulged Loops:

Synthesized probe sequences on the DNA microarray surface. Red letters highlight the additional loop bases.

a) Poly-C-Loops

loop position	loop length	Probe sequence	length (bases)
PM Probe		3’-TTCAATACTACTCATAATTACCAACAACATTAC-5’	33
9	1	3’-TTCAATACTCACTCATAATTACCAACAACATTAC-5’	34
9	2	3’-TTCAATACTCCACTCATAATTACCAACAACATTAC-5’	35
⋮	⋮	3’-TTCAATACTCC...ACTCATAATTACCAACAACATTAC-5’	⋮
9	13	3’-TTCAATACTCCCCCCCCCCCCCACTCATAATTACCAACAACATTAC-5’	46
16	1	3’-TTCAATACTACTCATACATTACCAACAACATTAC-5’	34
16	2	3’-TTCAATACTACTCATACCATTACCAACAACATTAC-5’	35
⋮	⋮	⋮	⋮
16	13	3’-TTCAATACTACTCATACCCCCCCCCCCCCATTACCAACAACATTAC-5’	46
23	1	3’-TTCAATACTACTCATAATTACCACACAACATTAC-5’	34
23	2	3’-TTCAATACTACTCATAATTACCACCACAACATTAC-5’	35
⋮	⋮	⋮	⋮
23	13	3’-TTCAATACTACTCATAATTACCACCCCCCCCCCCCCACAACATTAC-5’	46

b) Random Loop Sequences

loop position	loop length	Probe sequence	length (bases)
PM Probe		3’-TTCAATACTACTCATAATTACCAACAACATTAC-5’	33
9	1	3’-TTCAATACTCACTCATAATTACCAACAACATTAC-5’	34
9	2	3’-TTCAATACTCAACTCATAATTACCAACAACATTAC-5’	35
9	3	3’-TTCAATACTCAGACTCATAATTACCAACAACATTAC-5’	36
9	4	3’-TTCAATACTCAGTACTCATAATTACCAACAACATTAC-5’	37
9	5	3’-TTCAATACTCAGTCACTCATAATTACCAACAACATTAC-5’	38
⋮	⋮	3’-TTCAATACTCAGTC...ACTCATAATTACCAACAACATTAC-5’	⋮
9	13	3’-TTCAATACTCAGTCAGTCAGTCACTCATAATTACCAACAACATTAC-5’	46
16	1	3’-TTCAATACTACTCATACATTACCAACAACATTAC-5’	34
16	2	3’-TTCAATACTACTCATACAATTACCAACAACATTAC-5’	35
⋮	⋮	⋮	⋮
16	13	3’-TTCAATACTACTCATACAGTCAGTCAGTCATTACCAACAACATTAC-5’	46
23	1	3’-TTCAATACTACTCATAATTACCACACAACATTAC-5’	34
23	2	3’-TTCAATACTACTCATAATTACCACAACAACATTAC-5’	35
⋮	⋮	⋮	⋮
23	13	3’-TTCAATACTACTCATAATTACCACAGTCAGTCAGTCACAACATTAC-5’	46

Results:

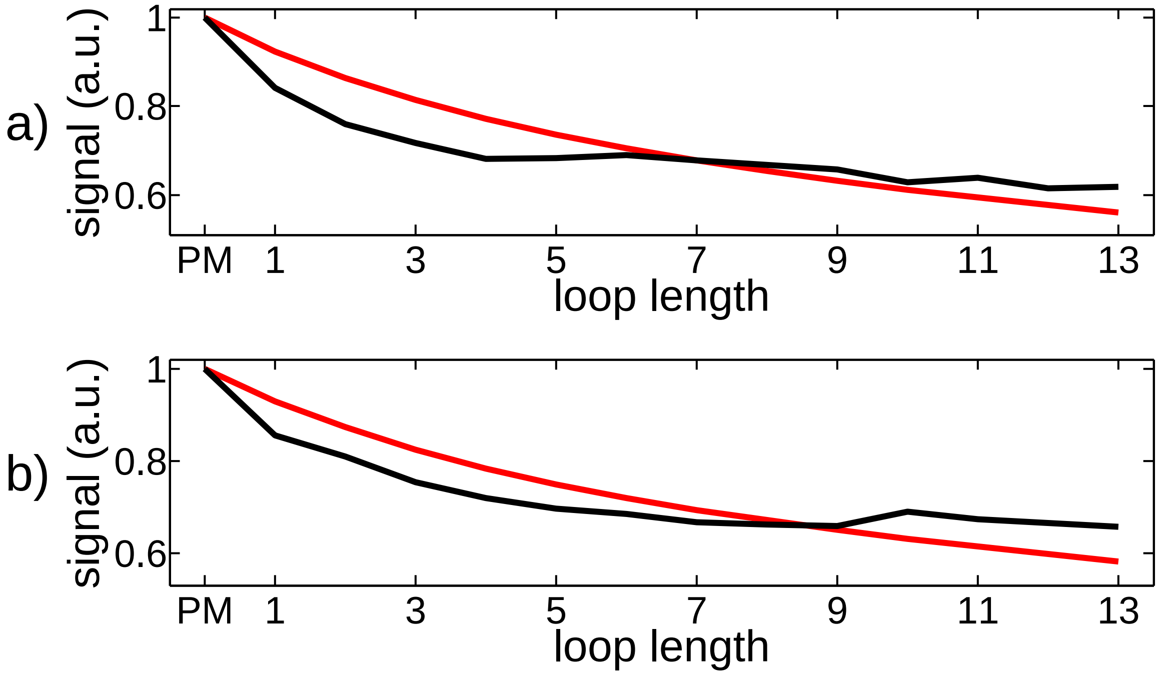

Fluorescent signals as a function of loop length after averaging over all loop positions and comparison to the prediction. Symbols: experimental signals, black solid line; predicted signals, red solid line. **a)** Poly-C-loops. **b)** Random loops.
