# Supplementary material for: Stability of double-stranded oligonucleotide DNA with a bulged loop: a microarray study
Source: BMC Biophys. 2011 Dec 13;4:20. doi: 10.1186/2046-1682-4-20 (PMC3262748; doi:10.1186/2046-1682-4-20)
Supplement: Additional file 3 — Influence of the number of MMs on the fluorescent signal. In order to reproduce our experimental data, it is sufficient to consider up to 3 synthesis-related defects in the zipper model. We confirm this by measuring the fluorescent signals of probes with 1 to 4 MMs. MMs are incorporated into the PM probe motif at 8 given positions resulting in 162 different probe sequences. To generate the MMs, we replace the bases at these specific positions with a thymine base (or with an adenine base, if a thymine base is already present at the specific position). After categorizing the probes into groups according to their number of MMs, we calculate the average signal of each group and plot it against the number of MMs (PM signal is set to 1, background signal is set to 0). Based on this data, we can estimate the error caused by neglecting probes with more than 3 synthesis defects: the error ≪ 4%, smaller than the experimental error. [file 2046-1682-4-20-S3.PDF]

Supplementary Material
Additional File 3

"Stability of double-stranded oligonucleotide DNA with a bulged loop: a microarray study"

Christian Trapp, Marc Schenkelberger and Albrecht Ott

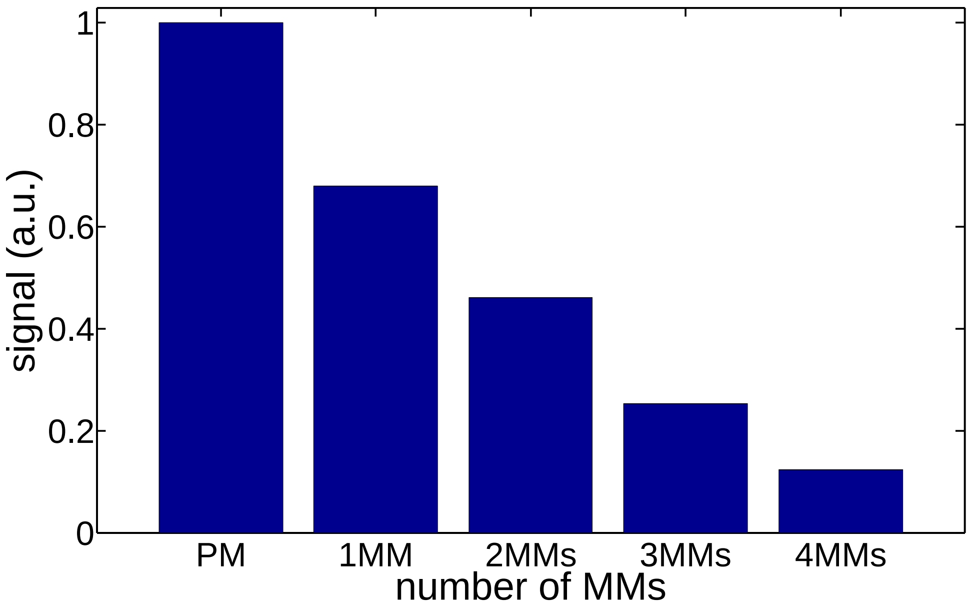

Mean intensity of features as a function of number of MMs in the probe sequence (PM signal is set to 1, background signal set to 0). Signal intensity decreases monotonically with the number of MMs. The mean intensity of a duplex with 4 MMs is only about 12% of the PM signal. Since 4MMs or more only account for about 30% of the synthesized probes, it is not necessary to include probes with more than 3 synthesis defects into the numerical calculation (numerical error$<< 4\%$).
