# Supplementary material for: Stability of double-stranded oligonucleotide DNA with a bulged loop: a microarray study
Source: BMC Biophys. 2011 Dec 13;4:20. doi: 10.1186/2046-1682-4-20 (PMC3262748; doi:10.1186/2046-1682-4-20)
Supplement: Additional file 4 — Hybridization signals resulting from Zdouble zipper and comparison to Zextended,right + Zextended,left. We compare the calculated hybridization signals resulting from Zdouble zipper to the signals resulting from. Zextended,right + Zextended,left.The predicted hybridization signals are similar in shape but differ regarding absolute values. In the figure, the scaling factor C, which relates the predictions to the absolute signal intensities of the experiments, has been changed to 3 in case of Zdouble zipper, compared to C = 1.5 · 10-3 throughout this study. [file 2046-1682-4-20-S4.PDF]

Supplementary Material
Additional File 4

"Stability of double-stranded oligonucleotide DNA with a bulged loop: a microarray study"

Christian Trapp, Marc Schenkelberger and Albrecht Ott

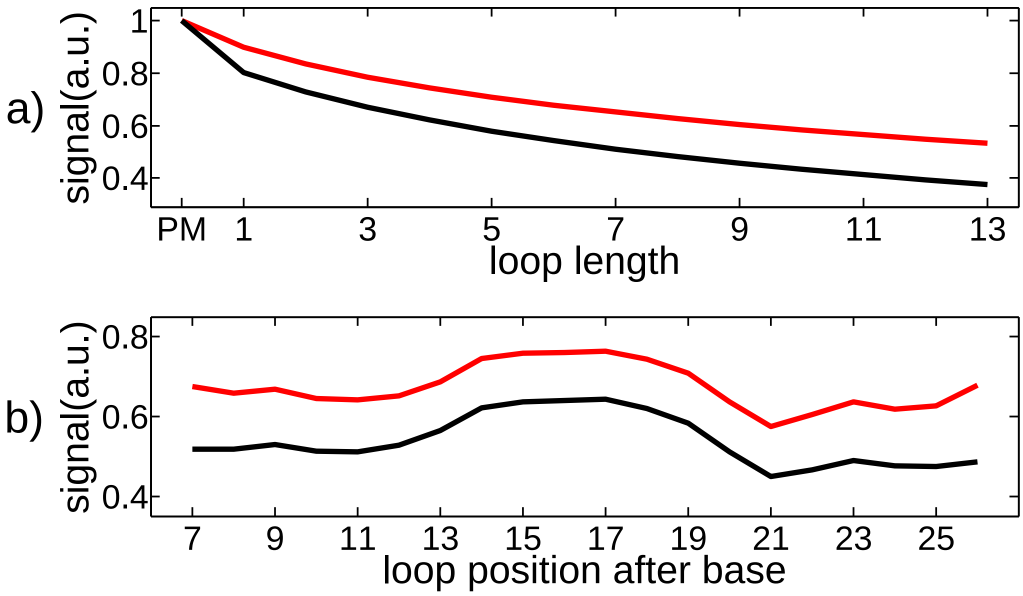

Symbols: red solid line, hybridization signals resulting from $Z_{extended,right} + Z_{extended,left}$ ($C = 1.5 \cdot 10^{-3}$ as used throughout the study); black solid line, hybridization signals resulting from $Z_{double\ zipper}$ ($C = 3$). **a)** Calculated hybridization signals as a function of loop length after averaging over all loop positions. **b)** Calculated hybridization signals as a function of loop position after averaging over all loop lengths. The approximate shape of the hybridization signals is similar but they differ in absolute values. In particular C, which relates the predictions to the absolute signals of the experiment, had to be increased by 3 orders of magnitude to reach the approximate dimension of the experimental values in the case of $Z_{double\ zipper}$. Hybridization temperature is 317 K.
